# Combining conventional QTL analysis and whole-exome capture-based bulk-segregant analysis provides new genetic insights into tuber sprout elongation and dormancy release in a diploid potato population

**DOI:** 10.1038/s41437-021-00459-0

**Published:** 2021-07-30

**Authors:** Sanjeev Kumar Sharma, Karen McLean, Richard J. Colgan, Debbie Rees, Stephen Young, Mads Sønderkær, Leon A. Terry, Colin Turnbull, Mark A. Taylor, Glenn J. Bryan

**Affiliations:** 1 Cell and Molecular Sciences, The James Hutton Institute, Dundee, United Kingdom; 2 Natural Resources Institute, University of Greenwich, Kent, United Kingdom; 3 Department of Chemistry and Bioscience, Aalborg University, Aalborg, Denmark; 4 Plant Science Laboratory, Cranfield University, Bedfordshire, United Kingdom; 5 Department of Life Sciences, Imperial College London, London, United Kingdom

**Keywords:** Genetic markers, Next-generation sequencing, Plant breeding, Agricultural genetics, Genetic mapping

## Abstract

Tuber dormancy and sprouting are commercially important potato traits as long-term tuber storage is necessary to ensure year-round availability. Premature dormancy release and sprout growth in tubers during storage can result in a significant deterioration in product quality. In addition, the main chemical sprout suppressant chlorpropham has been withdrawn in Europe, necessitating alternative approaches for controlling sprouting. Breeding potato cultivars with longer dormancy and slower sprout growth is a desirable goal, although this must be tempered by the needs of the seed potato industry, where dormancy break and sprout vigour are required for rapid emergence. We have performed a detailed genetic analysis of tuber sprout growth using a diploid potato population derived from two highly heterozygous parents. A dual approach employing conventional QTL analysis allied to a combined bulk-segregant analysis (BSA) using a novel potato whole-exome capture (WEC) platform was evaluated. Tubers were assessed for sprout growth in storage at six time-points over two consecutive growing seasons. Genetic analysis revealed the presence of main QTL on five chromosomes, several of which were consistent across two growing seasons. In addition, phenotypic bulks displaying extreme sprout growth phenotypes were subjected to WEC sequencing for performing BSA. The combined BSA and WEC approach corroborated QTL locations and served to narrow the associated genomic regions, while also identifying new QTL for further investigation. Overall, our findings reveal a very complex genetic architecture for tuber sprouting and sprout growth, which has implications both for potato and other root, bulb and tuber crops where long-term storage is essential.

## Introduction

Potato, the world’s most important non-cereal food crop, is a highly heterozygous polyploid outbreeding crop species. There has been significant recent progress in development of tools for linkage mapping and quantitative trait loci (QTL) analysis in tetraploid potato (Hackett et al. 2014; Hackett et al. 2013). Despite such advances, the majority of potato trait genetic analysis is still performed using crosses between heterozygous diploid parents (Bonierbale et al. 1988; Bryan et al. 2002; van Os et al. 2006). In fact, there has been a very recent move towards establishing diploid hybrid breeding in potato for propagation through true potato seeds, a concept that is gaining increasing momentum and which stands to change potato breeding radically (Bachem et al. 2019). Despite the recent availability of greatly improved tools and resources, such as dense SNP platforms, complex trait analysis in potato remains a challenging activity, although the publication of the potato genome (Potato Genome Sequencing Consortium 2011) now makes it possible to use genetic information to adopt candidate gene approaches for trait gene identification. An example of this is the recent identification of a HEAT SHOCK COGNATE 70 (*HSC70*) gene conferring heat tolerance in potato (Trapero-Mozos et al. 2018).

Potato tubers undergo a period of endodormancy after maturation and this inherent feature of tuber biology is one of the key determinants of tuber post-harvest storage life. The length of tuber dormancy differs among potato cultivars (Bamberg 2010; Bogucki and Nelson 1980; Vanittersum 1992). Tuber dormancy is important economically as it dictates, to a very large extent, how long varieties can be stored before processing or sale in fresh markets (Gebhardt et al. 2014). Premature dormancy release in tubers during storage is accompanied by significant deterioration in product quality (Sonnewald 2001). The potato processing industry currently controls sprouting by using chemical sprout suppressants and/or by storing tubers at low temperatures. However, in the EU, authorisation of products containing the most effective synthetic chemical inhibitor Chlorpropham (CIPC) for controlling sprouting were withdrawn from January 2020 (https://eur-lex.europa.eu/eli/reg_impl/2019/989/oj). Low temperature storage does suppress tuber sprouting in storage but also results in an undesirable accumulation of reducing sugars, which in turn increases both acrylamide forming potential and the incidence of browning in processed products cooked at high temperature (Paul et al. 2016). In addition, the consequential increase in energy use associated with low temperature storage has adverse impacts on the environment (Paul et al. 2016), and furthermore, may be prohibitively expensive in developing countries that are undergoing increased potato production. Besides the need to develop new storage strategies for potato, breeding potato cultivars with extended or otherwise modified (e.g. reduced for regions that employ multiple cropping cycles) dormancy or sprout vigour is desirable (Alamar et al. 2017). Starting with Simmonds (1964), the majority of genetic studies on tuber dormancy to date have been conducted at the diploid level (Freyre et al. 1994; Simko et al. 1997; van den Berg et al. 1996). QTL effects have been detected on several potato chromosomes but there has been virtually no progress in identifying the genes that exert genetic control of potato tuber dormancy and sprouting, partly due to the poor resolution of genetic maps that were used for work performed prior to the availability of modern marker technology platforms.

The aim of the current investigation was to dissect the genetic architecture of tuber sprout elongation using a large diploid mapping population (06H1) genotyped using a potato single-nucleotide polymorphism (SNP) array (Felcher et al. 2012; Hamilton et al. 2011). Tubers from 249 clones of the population were assessed for sprout growth in storage at six time-points over two consecutive growing seasons. In addition to a conventional linkage map-based QTL analysis, bulk-segregant analysis (BSA) using whole-exome capture (WEC) sequencing of ‘low’ and ‘high’ sprout-growth pools has been used for fine mapping of QTL and the identification of candidate genes underlying them.

## Materials and methods

### Plant material and field trials

A diploid mapping population (06H1) comprising a full-sibling progeny from a cross between two highly heterozygous diploid potato clones ‘HB171(13)’ (female parent) and ‘99FT1b5’ (male parent) was used in the current study. Both 06H1 parental clones are derived from crosses between Group Phureja and Tuberosum diploids as described in Prashar et al. (2014). A linkage map involving 186 individuals from 06H1 has been published (Prashar et al. 2014), alongside a QTL analysis of tuber shape and eye depth. For this study, we extended the linkage map to include 249 progeny clones phenotyped for tuber dormancy/sprout-growth-related traits. Field trials involving 06H1 parental and progeny clones were carried out as 8 plant plots per genotype, replicated twice using randomized complete block designs during two growing seasons (2013–2014) at Balruddery Farm, near Dundee in Scotland. All field trials were conducted following standard agronomic practices for fertilizer, pesticide applications and desiccation prior to harvest. No sprout suppressants were applied either pre- or post harvest.

### Phenotypic evaluation

Over two successive years (2013, 2014), the 249 clones from the 06H1 population plus the two parental clones were harvested into net bags (~10 tubers per plot), from two replicated field trial plots. Samples were sent by overnight courier to the Natural Resources Institute’s Produce Quality Centre, based at the East Malling Trust Estate in Kent, United Kingdom. After arrival tubers were placed into paper bags to exclude light and stored at 10 °C. Tubers were removed from store after 4 days and the apical buds were examined under a stereo microscope (Wild Heerbrugg, Switzerland), the proportion of dormant buds and tubers undergoing bud emergence were scored, and the length of the longest sprout was measured (mm) using electronic calipers (RS Components Ltd, Northants, UK). Repeat assessments on sprout length were made every 14 days (i.e. 4, 18, 32, 46, 60 and 74 days) for the two trial years. For each time-point, means across the ten assessed tubers for each replicate and clone were used in all subsequent analyses.

A General Analysis of Variance (ANOVA) was performed for each trait in each year using Genstat 19th Edition (VSN International 2019) using the mean phenotype for each genotype and replicate (two field replicates per genotype). Genotypic and residual mean square (MS) values from the ANOVA were used to calculate the overall genotypic variance (*σ*_*g*_^2^), the environmental variance (*σ*_*e*_^2^), broad-sense heritability (*H*^2^) of each trait. The broad-sense heritability (*H*^2^) of clone means was estimated as follows**:**

$$H^2 = \sigma _g^2/\left( {\sigma _g^2 + \left( {\sigma _e^2/2} \right)} \right)$$

where ***σ***_*g*_^2^ = (genotypic MS − residual MS)/2 (2 represents the number of reps) and ***σ***_*e*_^2^ = residual MS.

### Phenotypic data analysis

Data were fitted using a Gompertz plant growth model (Gompertz 1825; Winsor 1932; Zeide 1993), where the relative growth rate declines exponentially over time. The model assumes that an inflection point of the curve occurs at 0.368 of the maximal biomass. Due to the limited resources of the tuber and that tubers were stored in the dark at 10 °C, it can be assumed that an asymptotic growth rate and final length of emerging sprouts will be reached (Paine et al. 2012). A value of 90 mm was considered as the pre-set value for the asymptote sprout length and the data were fitted using a non-linear model (Bates and Chambers 1992; Bates and Watts 1988) to fit BETA (*β*) and KAPPA (κ) to the data using the equation as expressed below:

$${{{\boldsymbol{y = ae}}}}^{ - e^{\left( {\beta - \kappa x} \right)}}$$

where ***y*** is tuber sprout length in ‘mm’; ***x*** is time in ‘days’; ***a*** is asymptote for the growth curve, ***β*** sets the end of the dormancy period before growth of buds starts and ***κ*** controls rapid growth rate. The point of inflection of the curve reaches at 0.368**a**. The model fitting was implemented using the R statistical package (R Core Team 2016).

### Linkage and QTL mapping

The 06H1 parental and progeny clones were genotyped using Illumina Infinium 8k Potato SNP Array (Felcher et al. 2012; Hamilton et al. 2011) and the linkage map reported by Prashar et al. (2014) was extended to include a total of 249 individuals and 3052 marker loci using JoinMap4.1 (Van Ooijen 2011). Means for each time-point trait for all progeny clones for each year were calculated and used for the QTL analysis as well as bulk selection for WEC analysis. QTL mapping was performed using MapQTL^®^ 6.0 (Van Ooijen 2009) and Genstat 19th Edition (VSN International 2019). The non-parametric Kruskal–Wallis (KW) test supported in MapQTL version 6.0 was performed initially. In the KW method, a single-marker analysis is used to test the association of a marker with the trait at significance *P* ≤ 0.001. The identified QTL regions were further explored by using single-trait-single-environment QTL analysis using Genstat, treating each year’s data as separate datasets. This was done using simple interval mapping (SIM) followed by composite interval mapping (CIM), controlling the effects of chromosomes onto the QTL being tested and so increasing the precision of QTL detection (Zeng 1994). The QTL model fitted to the data was as follows:

$$y_i = \mu + {\Sigma}_{l \in L}\left( {x_{il}^{add}\alpha _l^{add}+ x_{il}^{add2}\alpha _l^{add} + x_{il}^{dom}\alpha _l^{dom}} \right) + G_i$$

where ***y***_*i*_ is the trait value of genotype *i*, ***x***_*il*_^*add*^ are the additive genetic predictors for maternal genotype *i* at locus *l*, ***x***_*il*_^*add2*^ are the additive genetic predictors for paternal genotype *i* at locus *l*, and ***α***^*add*^ and ***α***^*add2*^ are the associated effects, and ***x***_*il*_^*dom*^ are the dominance genetic predictors, and ***α***_*l*_^*dom*^ are the associated effects. Genetic predictors are genotypic covariables that reflect the genotypic composition of a genotype at a specific chromosome location (Lynch and Walsh 1998). ***G***_*i*_ is the residual unexplained genetic and environmental variation, which is assumed to follow a Normal distribution with mean 0 and variance *σ*^2^. For these analyses, the Genstat procedures QSQTLSCAN for candidate QTL identification, QSBACKSEL for QTL selection and QSESTIMATE for QTL model fitting were used.

Estimation of the four QTL genotype means at each of the QTLs detected for time-point t46 was calculated using the following models in GenStat: AC: mu − a1 − a2 + d, AD: mu − a1 + a2 − d, BC: mu + a1 − a2 − d, BD: mu + a1 + a2 + d; where mu is overall mean (constant in the model), a1 = additiveP1, a2 = additiveP2 and d = dominance effect. The additive effects were calculated as [(AC + AD) − (BC + BD)] for female parent, [(AC + BC) − (AD + BD)] for male parent, and [(AC + BD) − (AD + BC)] for interaction effects.

### WEC library construction on bulked samples and sequencing

Genomic DNA was extracted from young leaf tissue from individual 06H1 field-grown plants including parents using the Qiagen DNeasy Plant Maxi Kit (Qiagen) and quantified using the Quant-iTTM PicoGreen^®^ dsDNA Assay Kit (Invitrogen, San Diego, CA). The 20 most extreme clones from t46 sprout-growth measurements were selected for incorporating into ‘high’ and ‘low’ sprouting bulks from which equimolar DNA pools were prepared. Individual 06H1 clones comprising these pools are detailed in Table S1. These pooled samples as well as the 06H1 parents were then processed for preparing WEC libraries using Roche NimbleGen SeqCap EZ platform. Potato exome capture regions were mainly designed using PGSC (Potato Genome Sequencing Consortium 2011; Sharma et al. 2013) gene annotations and further supplemented by the additional potato protein-coding genes reported by the International Tomato Annotation Group (Tomato Genome Sequencing Consortium 2012). Regions matching to the organellar genomes (http://solanaceae.plantbiology.msu.edu/pgsc_download.shtml) were excluded from the probe design. The resulting set of target non-redundant genomic regions comprised 60.2 Mb and was used to design liquid capture probes by Roche Nimblegen following their standard protocols.

The WEC sequencing libraries were prepared using the designed capture probes according to the Roche NimbleGen SeqCap EZ Library preparation protocol. A total of 100 ng of genomic DNA for each sample was fragmented to an average size range of 180–220 bp using Covaris M220 (Covaris Ltd., Woodingdean, Brighton, UK) shearing conditions. Fragmentation quality was assessed by running 1 μl of the processed sample on a Bioanalyzer High-Sensitivity DNA chip (Agilent technologies). The remainder of each sample was subjected to end repair, A-Tailing, adapter ligation and fragment size-selection; all steps performed using the KAPA Library preparation kit (Roche Diagnostics Corporation, USA). Fragment-size distribution of all libraries was re-assessed using Bioanalyzer High-Sensitivity DNA chip. Each size-selected library was subjected to pre-capture PCR with the cycling conditions, as follows: initial denaturation at 98 °C for 45 s; 9 cycles of 98 °C for 15 s, 60 °C for 30 s and 72 °C for 30 s; final extension at 72 °C for 1 min, and cooling to 4 °C until further use. The pre-capture PCR products were purified with AMPure XP beads (Beckman Coulter Ltd, UK) and analysed on a Bioanalyzer High-Sensitivity DNA chip. The yield of each library was quantified using the Qubit^®^ dsDNA High Sensitivity Assay Kit (Life Technologies). Two sets of composite samples for hybridization were prepared—one for parental clones and the other for bulk samples. In each set, sample pairs were multiplexed using Illumina Truseq DNA LT adapters. Equal amount (500 ng) of amplified sample libraries for each set was pooled into a composite sample (1 µg in total), which was hybridized with potato WEC oligomers at 47 °C for ~65 h. The hybridized library was washed, captured-DNA recovered and amplified using the similar PCR conditions as pre-capture PCR but for 14 cycles. The final PCR product was cleaned up using AMPure XP Beads and assessed for quality and fragment-size distribution using Bioanalyzer High-Sensitivity DNA chip. Captured-DNA quantification was performed using Qubit^®^ dsDNA High Sensitivity Assay Kit. In parallel, as a control sample, WEC library was also constructed for the doubled monoploid *Solanum tuberosum* group Phureja DM1-3 516 R44 clone (Lightbourn and Veilleux 2007), hereafter referred to as DM, used for sequencing the reference potato genome (Potato Genome Sequencing Consortium 2011). The parent clones and control DM sample hybridized captures were sequenced together on a single lane while the pooled bulk samples hybridized capture was sequenced on a separate lane of the Illumina HiSeq 4000 platform to generate 150 bp paired-end sequence reads. Sequencing was performed at the Edinburgh Genomics (Edinburgh, UK) facility.

### Read mapping and variant detection

WEC paired-end reads obtained for 06H1 parents, two dormancy bulks and the control DM sample were processed for adapter trimming and quality filtering using Trimmomatic (Bolger et al. 2014). The processed reads were mapped to the potato reference pseudomolecules (Potato Genome Sequencing Consortium 2011; Sharma et al. 2013) using Bowtie2 (Langmead and Salzberg 2012). Alignments showing PCR duplicates, multiple mappings or not having proper pairs were filtered. Quality alignments were further processed through local realignment and base quality recalibration tools followed by SNP discovery using GATK4 (DePristo et al. 2011). WEC data for each of the 06H1 parent and bulk samples were processed separately.

### Bulk-segregant analysis (BSA)

BSA was performed using WEC SNPs identified in the two parental clones. Only the SNP base positions displaying a minimum coverage (read depth) of 10 across both parents as well as bulks were used in the analysis. For these SNP locations, base distribution from ‘low’ and ‘high’ tuber sprouting bulks was compared with each other using Chi-squared test and, for ascertaining significance in allelic variation at the compared SNP positions, *Q* values were recorded. BSA was performed ‘reciprocally’ i.e. in one comparison allele frequencies in the ‘low’ bulk were considered as ‘observed’ and in ‘high’ bulk as ‘expected’ for performing the Chi-squared test and vice-versa. Average *Q*-values (AQVs) and relative skewness (RS), for assessing non-random distribution of allele frequencies, between significant and non-significant marker regions were calculated in window sizes of 500 WEC-derived SNPs (subsequently referred to as ‘marker-bin’) in steps of 50 markers along each chromosome. For declaring significance, a 5% false discovery rate (FDR) threshold on AQV calculated for each marker-bin was applied. AQV cut-off limits corresponding to the set (5%) FDR threshold were calculated separately for each individual chromosome. Marker-bins surpassing these AQV cut-off limits were considered to contain SNPs displaying significant differences in allelic frequencies between the two bulks. Differences in SNP-allele frequencies between the two bulks were visualised by plotting RS and AQV along the chromosomes using Circos (Krzywinski et al. 2009). BSA was performed as reported by Kaminski et al. (2015).

## Results

### Linkage map construction

The linkage map of the 06H1 population published by Prashar et al. (2014) was expanded to include a total of 249 individuals. The inclusion of ‘nearly co-segregating’ markers omitted from the previous study led to an increase in mapped SNP markers from 2157 to 3076, of which 3052 were uniquely mapped (Fig. S1). The map statistics for individual chromosomes and the overall linkage map are given in Table S2. The new combined map length of the 12 linkage groups (778.4 cM) compares well with a total map length of 754 cM in the previously published map (Prashar et al. 2014), with an increase in the overall map length of only 3.2%, suggesting that both linkage maps are highly accurate.

### Tuber sprout-growth patterns

From a physiological perspective, it has been argued that tuber dormancy commences at tuber initiation, when activity in the apical meristem in the stolon apex is suspended (Claassens and Vreugdenhil 2000). However, from a practical perspective, it is extremely difficult to measure tuber initiation accurately in a large population of field-grown genotypes. More practical measures are the time from harvest to initiation of sprout growth (as a proxy for tuber dormancy period) and rate of sprout growth, and these parameters, relevant for assessing the storage characteristics of a crop, were assessed in this study.

**Fig. 1:**
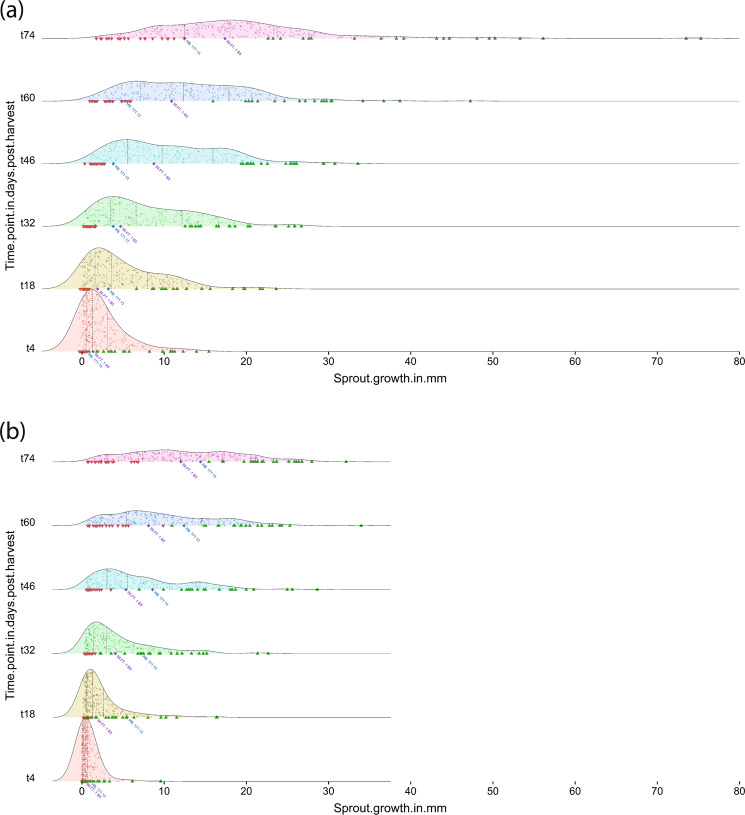
06H1 density plots for all time-point sprout-growth traits. Blue and Purple-labelled ‘diamond’ symbols represent female ‘HB171(13)’ and male ‘99FT1b5’ parent values. Clones included in ‘low’ and ‘high’ sprout-growth rate bulks—selected on the basis of time-point t46 observations—are shown in downward (red) and upward (green) pointing triangles, respectively. **a** 2013; **b** 2014.

**Fig. 2:**
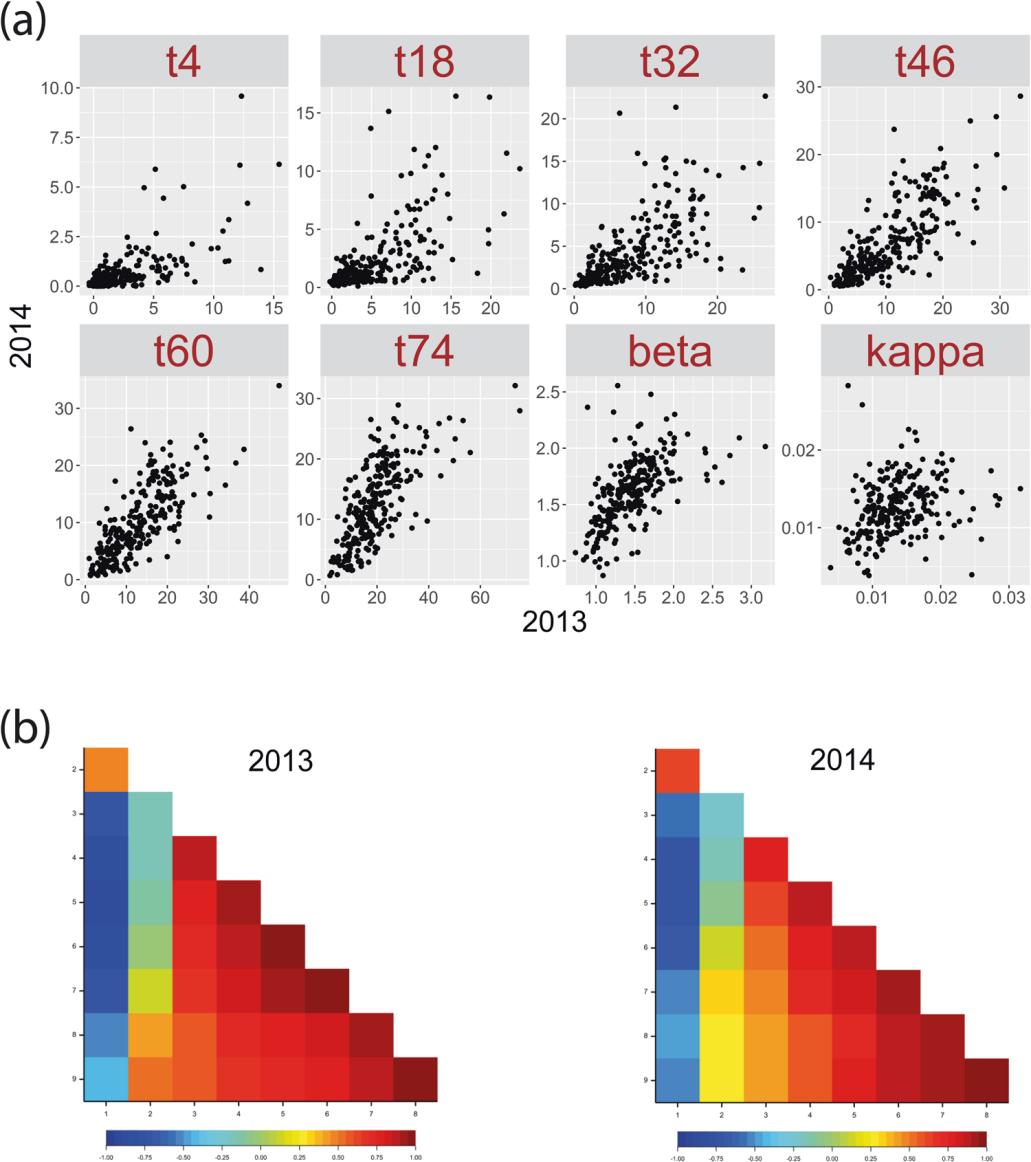
Comparison of sprout growth data from two assessment years and trait correlations within each assessment year. **a** Scatter plots of mean trait data from 249 ‘06H1’ progeny clones for all time-points for the 2 years assessed (2013 vs. 2014); **b** Correlation heat maps of eight tuber dormancy and sprout-growth traits assessed in each year. Traits are: (1) t14; (2) t18; (3) t32; (4) t46; (5) t60; (6) t74; (7) BETA, (8) KAPPA. Correlation coefficients (*r*) are provided in Table S3.

The tuber sprout-growth parameters were measured on sets of 10 tubers per clone per field replicate, and values used for further analysis represent the mean for each replicate and genotype for each year. The 06H1 population exhibited a relatively low average level of dormancy, presumably due to the use of two Phureja clones showing extremely low dormancy as grandparents, each parent being a Phureja-Tuberosum ‘hybrid’. The parental clones generally showed intermediate levels of dormancy and sprout growth as shown by density plots of the time course sprout-growth data for each season (Fig. 1a, b). In year 1 (2013), the male parent showed a higher rate of sprout growth (2.30 times at t46) compared with the female parent except for one early time-point (t18), while in year 2 (2014), a reverse trend was revealed, with the female parent displaying a faster sprout-growth rate (1.61 times at t46). The density plots show a gradual broadening of the range of phenotypic values with increasing time since harvest. Trait mean values for the population generally showed high correlations between the 2 year’s data (Fig. 2 and Table S3). The BETA and KAPPA traits are modelled ‘derived’ traits, estimating the dormancy period before growth of buds starts and initial sprout-growth rates, respectively, so it is perhaps not surprising these show lower correlations than the directly measured traits. The 06H1 population tended to display longer dormancy in 2014 (indicated by the shape of the graph in Fig. 2, and presence of more clones with sprout lengths near zero at the earlier time-points, Fig. 1b). The rate of sprout growth was similar until 60 days, but at later time-points, the data indicate that sprout-growth rates were higher in 2013 than 2014. The correlation data for the eight sets of trait means within each of the two assessment years are shown as heat maps in Fig. 2b. Generally, as expected, the correlations between the ‘time-point’ sprout length traits are very high and positive (>>0.5). The correlations at the earlier time-points are slightly weaker in 2014. For the ‘BETA’ trait, a measure of the dormancy period before bud growth, the data show, as expected, a strong tendency towards a high negative correlation with sprout-growth traits. In contrast, the ‘KAPPA’ trait shows a very weak correlation with all traits except the later time-points (t74), where there are stronger positive correlations. The weak correlations at early time-points could be attributed to the dependence of sprout length both on length of dormancy period and subsequent sprout vigour.

### Variance components and broad-sense heritabilities for sprout-growth data

For each time-point and modelled trait, a simple ANOVA was performed. The variance components (Genotype, Residual) are shown in Table 1. The genotypic variance was tested against the residual for each trait and all were highly significant (*P* < 0.001). These variances were used to calculate broad-sense heritabilities for each trait. It can be seen that the broad-sense heritabilities range from 0.7 to 0.9, signifying a high level of overall genetic control for the traits under study, perhaps also reflecting that mean trait values were the mean of measurements taken from 10 tubers per genotype per replicate.

**Table 1:** Variance components and broad-sense heritability estimates for all time-point traits as well as BETA and KAPPA.

Year	Trait	Genotypic variance	*σ*^2^_*e*_	*σ*^2^_*g*_	*H*^2^
2013	t4	15.94	4.42	5.76	0.72
	t18	45.11	8.93	18.09	0.8
	t32	67.46	10.25	28.605	0.85
	t46	92.87	14.95	38.96	0.84
	t60	111.94	15.69	48.125	0.86
	t74	233.84	28.45	102.695	0.88
	BETA	0.23	0.06	0.085	0.74
	KAPPA	0.000046	0.000013	0.0000165	0.72
2014	t4	2.46	0.52	0.97	0.79
	t18	17.33	2.63	7.35	0.85
	t32	35.94	5.27	15.335	0.85
	t46	62.7	8.63	27.035	0.86
	t60	78.69	13.57	32.56	0.83
	t74	89.12	14.32	37.4	0.84
	BETA	0.29	0.05	0.12	0.83
	KAPPA	0.00002	0.000006	0.000007	0.7

*σ*^2^*e* = environmental variance, *σ*^2^*g* = genotypic variance, *H*^2^ = broad-sense heritability.

### Identification of QTL for tuber sprouting

Initially, a non-parametric KW analysis was carried with MapQTL6 software using the phenotypic datasets for the two trial years and the updated linkage map in order to detect associations between markers and traits. The results of this analysis are shown in Table S4. For brevity, the prefix ‘solcap_snp’ is omitted from Infinium SNP names. Significant genetic effects were detected on five linkage groups, 2 (c1_9719, 12.84 cM), 3 (c2_17218, 3.27 cM and c2_52371, 19.83 cM), 4 (c1_11758, 38.97 cM), 5 (c2_8515, 60.76 cM) and 10 (c2_54862, 41.99 cM). The magnitude of the detected effects is generally quite small for the earlier time-points assessed (e.g. t4, t18), but is significantly larger at the final time-point examined (e.g. t74) as shown by the considerably higher *K* values. However, for the QTL on chromosome 3, *K* values are high at the start of sprout growth suggesting that QTL on this chromosome are largely impacting on dormancy break rather than sprout-growth rate alone. It is also clear that both parents are contributing allelic variation impacting on the various time-point traits, although for four of the six loci detected, it is a paternal marker (i.e. nn × np in JoinMap notation) that appears to be having the largest effect.

**Fig. 3:**
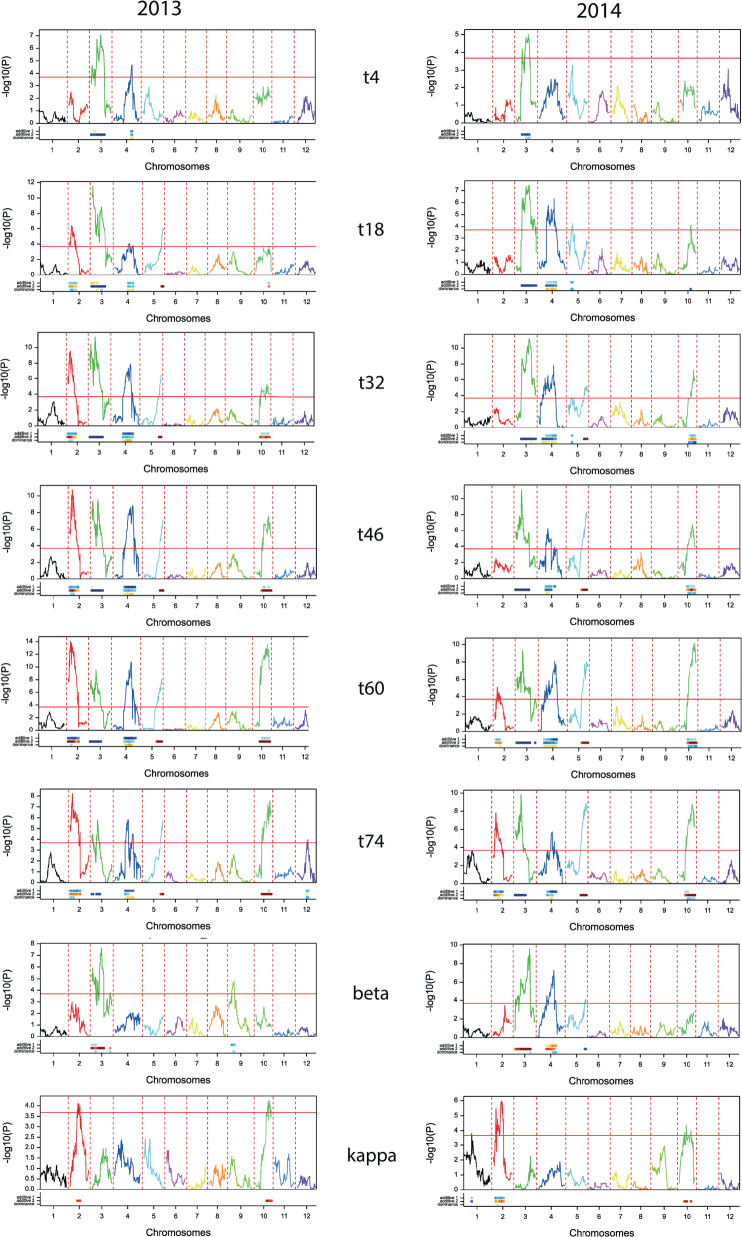
Composite interval mapping (CIM) QTL profiles for all time-points and modelled parameters BETA and KAPPA. Each box shows −log_10_*P* values plotted against chromosomal position for each of the 12 potato linkage groups for one trait. The −log_10_*P* value required for significance at the 1% level is indicated by the red horizontal line.

To further elucidate the genetic architecture of the selected traits, SIM and CIM in Genstat using a ‘single-environment-single-trait’ approach were also employed whereby each time-point trait and assessment year (2013 and 2014) were analysed separately for candidate QTL. Where possible, a QTL model was fitted after a suitable number of iterations. The routine DQSQTLSCAN (QSQTLSCAN procedure) was used initially with a genome-wide threshold of 0.01 (−log_10_*P* value of 4.176 at 1%) to select candidate QTL. Subsequently analysis was performed by using candidate QTL detected by DQSQTLSCAN as co-factors, extending the co-factor selection in subsequent iterations, which aimed to remove a co-factor from the model (loci selected by QSBACKSEL) if another QTL was evaluated within 10 cM. QTL additive and dominance effects and their standard errors were estimated for all QTL detected (using QSESTIMATE) and are shown for all six time-points, as well as the modelled parameters BETA and KAPPA in Table 2, with CIM-QTL profile plots being shown in Fig. 3. The results, which are generally in good agreement with those obtained using the non-parametric KW method, show a complex pattern of several unlinked loci on the same five linkage groups (2, 3, 4, 5 and 10) acting both positively and negatively on tuber sprout growth at the various QTL locations. A further small effect for BETA was detected on chromosome 9, but only in the first assessment year (Table 2). In several cases, effects on sprouting and dormancy phenotypes originate from both parents and each parent segregates for alleles impacting both positively and negatively on each trait, perhaps explaining why there is so much transgressive segregation in this population.

**Table 2:** Significant QTL effects detected by CIM for sprout growth at all six time-points (4, 18, 32, 46, 60 and 74 days post harvest) and Gompertz model parameters BETA and KAPPA.

Trait	Year	LG	Locus	Position (cM)	−log_10_(*P*)	Add. Eff (maternal)	s.e	Add. Eff (paternal)	s.e.	Dom. Eff	s.e.
t18	2013	2	c2_33973	13.5	6.23	−0.84	0.25	0.80	0.25	−0.72	0.25
t32	2013		c2_32421	13.79	8.9	−0.91	0.28	1.54	0.28	–	–
t46	2013		c2_33973	13.5	9.87	−1.01	0.32	1.84	0.31	–	–
t60	2013		c2_32421	13.79	11.11	−1.32	0.35	2.08	0.35	–	–
t60	2014		c2_33993	14.04	5.15	−0.84	0.3	1.16	0.29	–	–
t74	2013		c2_33993	14.04	9.78	−1.60	0.53	3.21	0.53	–	–
t74	2014		c2_33993	14.04	9.76	−1.42	0.33	1.66	0.32	–	–
KAPPA	2013		c2_51998	32.52	4.44	–	–	0.00	0	–	–
KAPPA	2014		c2_51998	32.52	7.11	0.00	0	0.00	0		
t4	2013	3	c2_53699	31.42	6.96	0.32	0.17	−0.96	0.17	–	–
t4	2014		c1_432	39.55	4.77	–	–	−0.33	0.07	–	–
t18	2013		c2_50372	3.46	4.81	0.65	0.28	−1.15	0.27	–	–
t18	2013		c2_55072	34.85	3.91	–	–	−1.19	0.28	–	–
t18	2014		c2_13987	41.71	8.81	0.38	0.16	−1.06	0.17	–	–
t32	2013		c1_6869	19.39	10.49	–	–	−2.09	0.29	–	–
t32	2014		c2_57254	43.32	10.83	–	–	−1.66	0.23	–	–
t46	2013		c1_6869	19.39	9.7	–	–	−2.23	0.32	–	–
t46	2014		c1_6875	19.28	10.39	–	–	−2.09	0.29	–	–
t60	2013		c2_57360	18.73	8.58	–	–	−2.39	0.36	–	–
t60	2014		c2_57360	18.73	8.89	–	–	−1.97	0.3	–	–
t60	2014		c2_578	60.44	2.86	–	–	−1.07	0.29	–	–
t74	2013		c2_57354	18.72	6.69	–	–	−2.90	0.53	–	–
t74	2014		c2_57360	18.73	9.75	–	–	−2.30	0.33	–	–
BETA	2013		c2_53699	31.42	6.84	–	–	0.13	0.02	–	–
BETA	2014		c2_57354	18.72	3.28	–	–	0.06	0.02	–	–
BETA	2014		c2_17770	48.93	3.66	–	–	0.07	0.02	–	–
t4	2013	4	c2_26741	58.77	4.6	−0.53	0.17	−0.48	0.16	0.47	0.17
t18	2013		c1_11211	46.45	5.34	−0.92	0.25	−0.78	0.25	0.53	0.25
t18	2014		c2_49997	48.22	6.49	−0.44	0.16	−0.57	0.16	0.57	0.16
t32	2013		c2_26741	58.77	7.46	−1.39	0.28	−0.75	0.28	0.96	0.28
t32	2014		c2_44609	16.79	2.19	–	–	−0.74	0.24	–	–
t32	2014		c2_49997	48.22	3.87	−0.88	0.25	−0.58	0.25	–	–
t46	2013		c1_8330	58.73	8.56	−1.81	0.32	−0.88	0.31	0.93	0.31
t46	2014		c1_15982	29.97	5.99	−0.87	0.28	−1.25	0.28	0.57	0.28
t60	2013		c2_26741	58.77	9.19	−1.99	0.36	−0.87	0.34	1.33	0.35
t60	2014		c2_26843	15.88	1.9	–	–	−0.75	0.33	0.71	0.29
t60	2014		c2_49997	48.22	4.95	−1.40	0.33	−0.79	0.34	–	–
t74	2013		c2_26741	58.77	8.78	−2.96	0.54	−1.71	0.52	1.65	0.53
t74	2014		c1_13603	44.68	6.19	−1.54	0.33	−0.80	0.33	–	–
BETA	2014		c2_21364	52.38	3.89	0.05	0.02	0.05	0.02	–	–
t18	2013	5	c1_1216	63.72	4.86	–	–	1.19	0.25	–	–
t18	2014		c2_22995	14.03	4.13	−0.51	0.17	−0.13	0.17	−0.54	0.17
t32	2013		c1_1216	63.72	6.66	–	–	1.58	0.28	–	–
t32	2014		c2_22995	14.03	4.98	−0.69	0.22	–	–	−0.82	0.23
t32	2014		c1_1077	63.37	7.2	–	–	1.25	0.21	–	–
t46	2013		c1_1216	63.72	7.24	–	–	1.85	0.31	–	–
t46	2014		c1_1077	63.37	6.75	–	–	1.59	0.28	–	–
t60	2013		c1_1216	63.72	7.28	–	–	2.14	0.35	–	–
t60	2014		c2_8522	59.42	9.07	–	–	1.99	0.3	–	–
t74	2013		c1_1250	63.73	4.88	–	–	2.50	0.52	–	–
t74	2014		c2_8210	62.17	9.05	–	–	2.15	0.32	–	–
BETA	2014		c1_1077	63.37	5.51	–	–	−0.08	0.02	–	–
BETA	2013	9	c2_13304	16.5	3.49	−0.09	0.02	–	–	–	–
t18	2014	10	c2_40763	36.25	4.21	−0.39	0.16	–	–	−0.61	0.16
t32	2013		c2_27795	44.4	5.74	−0.64	0.28	1.32	0.28	–	–
t32	2014		c1_13243	47.07	5.95	−0.52	0.21	0.78	0.21	−0.72	0.21
t46	2013		c2_27795	44.4	7.39	−0.69	0.31	1.75	0.31	–	–
t46	2014		c2_27795	44.4	7.18	−0.79	0.28	1.36	0.28	−0.75	0.28
t60	2013		c2_27795	44.4	9.06	−0.81	0.35	2.28	0.34	–	–
t60	2014		c2_27831	47.32	10.94	−0.65	0.29	2.01	0.29	−0.85	0.29
t74	2013		c2_27831	47.32	8.08	–	–	3.26	0.53	–	–
t74	2014		c2_27821	42.99	7.6	–	–	1.91	0.32	–	–
KAPPA	2013		c2_54444	41.49	4.6	–	–	0.00	0	–	–
KAPPA	2014		c2_32740	22.97	5.54	–	–	0.00	0	–	–

Data are presented in linkage group order with maternal and paternal additive effects presented separately, along with overall dominance effects.

The magnitude of the additive QTL effects shows a slight tendency to increase over time (Table 2 and Fig. 3). One key observation is that a higher overall proportion (62 of 95 in total) of the significant additive effects on tuber sprout growth originates from the male parent. Regarding the female parent, 29 of 33 additive effects on sprout growth are negative whereas the male parent (99FT1b5) exhibits a roughly equal number of positive and negative effects, 33 and 29, respectively. The additive effects observed show a tendency to be both negative (maternal) and positive (paternal) on chromosomes 2 and 10, mainly negative (paternal) on chromosome 3, negative on chromosome 4 (both maternal and paternal) and positive on chromosome 5 (paternal). There are rather few dominance effects, mainly one on chromosome 4 that increases sprout growth at most time-points, one on chromosome 5 that decreases sprout growth in 2014 at two time-points (t18 and t32), and another on chromosome 10 that also decreases sprout growth in 2014 at all time-points except t74. Generally, fewer significant QTL effects are detected using the estimated parameters from the Gompertz modelling. The only significant QTL detected for the KAPPA parameter are two miniscule positive effects on chromosomes 2 and 10 in both years, which is also in agreement with the KW analysis that only detected two slightly significant effects but only for the second assessment year. For the estimate of dormancy period, BETA, there are small but significant effects on chromosomes 3, 4, 5 and 9. Overall, there are some inconsistencies in map position of QTL effects within the same linkage group but generally the locations fall within a fairly narrow range. For example the additive effects on chromosome 2 locate to a narrow region between 13.5 and 14.0 cM, although the KAPPA effects maps to 32.5 cM. However, the QTL effects on chromosome 3 suggest two or more potential QTL at ~3.5, 18.7–19.3 and 31.4–60.4 cM at the different time-points. Chromosome 4 shows a similarly dispersed pattern of QTL effects although most of them appear between 44.7 and 58.8 cM. Chromosome 5 markers linked to QTLs are mostly located between 59.4 and 63.7 cM. Similarly, QTL effects on chromosome 10 are fairly tightly clustered between 43 and 47.3 cM. These findings go some way to explaining the highly complex nature of dormancy and sprout growth in potato, especially given that this complexity has been revealed in a diploid progeny, with a maximum of four alleles per locus. The approximate colocalization of QTL for the BETA trait, which should give an indication of time to dormancy break, on chromosomes 3, 4 and 5 with those for sprout growth, suggests that sprouting QTL effects on these chromosomes are more likely to be related to time to dormancy break and early sprout growth. The situation for KAPPA is less clear and the QTL effects detected, while significant, are extremely small.

**Fig. 4:**
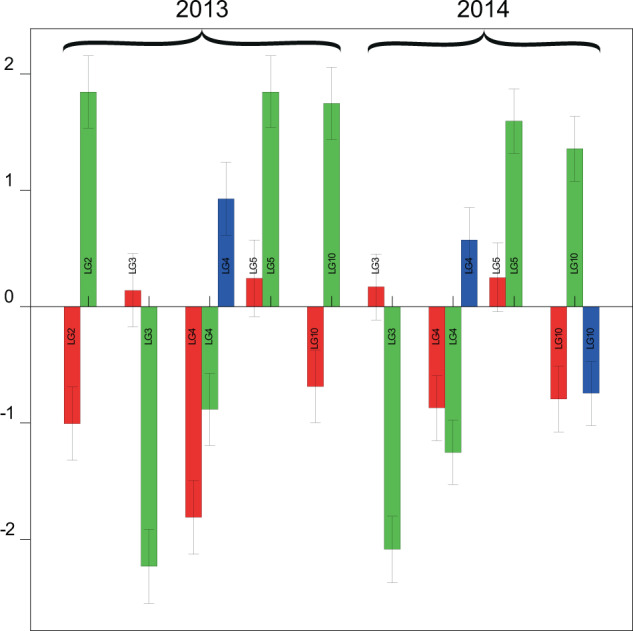
Bar chart showing magnitude and direction of additive and dominance effects of QTL detected at t46 (values given in Table 2). Error bars are indicated on each bar. The nine loci detected comprised five in 2013 [c2_33973 (LG2), c1_6869 (LG3), c1_8330 (LG4), c1_1216 (LG5), c2_27795 (LG10)], and four in 2014 [c1_6875 (LG3), c1_15982 (LG4), c1_1077 (LG5), c2_27795 (LG10)]. Red, green and blue bars illustrate maternal, paternal and dominance effects, respectively.

For time-point t46, a bar chart shows the magnitudes and standard errors of the significant additive and dominance effects on the five chromosomes (Fig. 4). For this time-point, we have also used the output from QSESTIMATE to calculate the four QTL genotype means at each of the QTLs detected. The QTL genotypic means and the maternal, paternal and dominance contributions are given in Table 3. Mean sprout growth at t46 was less in 2014 (6.94) than 2013 (10.31). The quite large maternal and paternal effects on chromosome 2 in 2013 were not observed in 2014. Otherwise the maternal and paternal additive effects for the four loci on chromosomes 3, 4, 5 and 10 are remarkably consistent in both magnitude and direction.

**Table 3:** Estimates of the QTL genotypic means and allelic effects at each of the QTLs detected for time-point t46.

Year	LG	cM	Marker	Mean	Genotypic means				Allelic effects		
				mu	ac	ad	bc	bd	Maternal	Paternal	Interaction
2013	2	13.5	c2_33973	10.31	9.47	13.16	7.46	11.15	4.02	−7.38	0
	3	19.39	c1_6869		12.4	7.94	12.68	8.22	−0.56	8.93	0
	4	58.83	c1_8330		13.93	10.31	8.46	8.54	7.24	3.53	3.71
	5	63.72	c1_1216		8.22	11.91	8.71	12.4	−0.97	−7.38	0
	10	44.4	c2_27795		9.25	12.74	7.88	11.37	2.74	−6.99	0
2014	3	19.39	c1_6869	6.94	8.86	4.68	9.2	5.02	−0.68	8.35	0
	4	29.97	c1_15982		9.64	5.99	6.75	5.39	3.49	5.01	2.29
	5	63.37	c1_1077		5.1	8.28	5.6	8.78	−1	−6.37	0
	10	44.4	c2_27795		5.63	9.83	5.54	6.76	3.17	−5.42	−2.98

### BSA on tuber sprout-growth pools using WEC

**Fig. 5:**
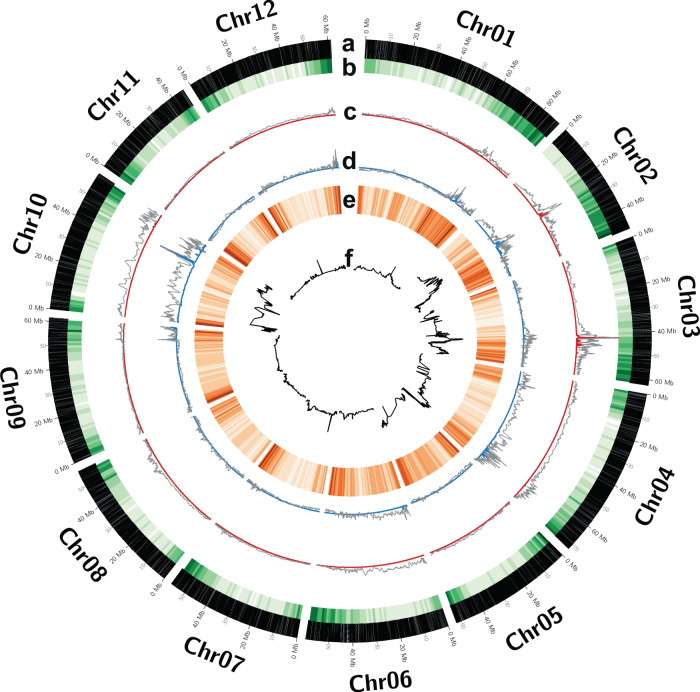
Bulk-segregant analysis (BSA) for comparing allele frequencies from ‘low’ and ‘high’ tuber sprouting bulks designed using t46 time-point trait. **a** Chromosomes, **b** gene density, **c** BSA: low sprout-growth rate bulk (observed) versus high sprout-growth rate bulk (expected); **d** BSA: high sprout-growth rate bulk (observed) versus low sprout-growth rate bulk (expected); **e** SNP density; **f** QTL detected by composite interval mapping (CIM) using t46 trait data. The terms ‘observed’ and ‘expected’ in (**c**) and (**d**) above refer to the assumptions set for using allele frequencies for performing the Chi-squared tests in reciprocal BSA analyses. Red and blue lines represent relative skewness (RS) measures while grey lines illustrate average-*Q*-values (AQV) estimates observed in the two reciprocal BSA comparisons for assessing non-random distribution of allele frequencies between the two bulks.

For performing BSA, 06H1 progeny clones displaying extreme low and high sprout growth were identified using the phenotypic data for the t46 trait. For construction of phenotypic bulks for WEC sequencing, DNA from progeny clones belonging to ‘low’ and ‘high’ sprout-growth bulks, 20 clones each (Table S1), were normalized and pooled in equimolar quantities. These normalized bulk samples, as well as DNA samples from parental clones, were subjected to WEC sequencing followed by variant discovery. The mean target coverage (read depth) across these samples ranged from 95 to 143 while the range for median target coverage (read depth) was 86–132. WEC probes were designed against the DM reference genome (Potato Genome Sequencing Consortium 2011). Therefore, to check the efficacy of the designed capture, the DM clone was also included as a control sample during the library preparation. Table 4 shows the summary statistics for total reads and sequencing coverage (read depth) achieved across all samples. SNP discovery for the female ‘HB171(13)’ and male ‘99FT1b5’ parental clones was performed separately yielding a total of 1112,418 and 1038,233 SNPs, respectively. A marker list containing 1032,795 SNPs was generated by combining SNP locations from both parental clones where SNPs were included only if their genomic positions were covered in both parents at the set (≥10x) sequencing coverage (read depth) regardless of the SNP genotypic states in the two clones. Positions in the parental marker list were used to statistically compare base coverage (read depth) distributions in ‘low’ and ‘high’ sprout-growth bulks using Chi-squared tests, and only for positions where the sequencing read depth in both bulks was more than or equal to 10. From this analysis, *Q* values for ascertaining significance in allelic variation at the compared SNP positions were recorded. AQVs and RS measures in a sliding window of 500 markers (marker-bin) were derived as described in ‘Materials and methods’. The AQV cut-off values, at 5% FDR threshold, derived using SNP *Q* values separately for each chromosome are detailed in Table S5. Marker-bins showing AQV higher than their respective chromosome cut-off limits were considered significant for the presence of non-random distribution of allele frequencies between the two bulks. The AQVs and RS estimates observed in the two reciprocal BSA comparisons are illustrated in Fig. 5 along with the results obtained from CIM for t46 time-point trait. The peaks as revealed from RS and AQV plots correspond to genomic regions showing non-random distribution of nucleotide frequencies between the two bulks potentially associated with the trait (t46) used for construction of bulks. From these, only those peaks, which comprised marker-bins exceeding FDR-derived AQV cut-off limits, in at least one of the reciprocal comparisons, were designated as BSA-QTL for tuber sprout growth. BSA-QTL were observed on chromosomes 1–8 and the regions spanning these QTL, with boundaries consolidated over the two reciprocal comparisons, are detailed in Table 5. Many of these QTL corroborated well with those obtained from CIM performed for t46 (Fig. 5f). Genomic coordinates for marker-bins with the highest AQV (top marker-bin) in BSA-QTL regions were derived and physical positions for the overlapping top marker-bins between the peaks originated from the two reciprocal comparisons were consolidated. These genomic coordinates corresponding to top marker-bins for each significant BSA-QTL are presented in Table 6. The smallest significant marker-bin (0.15 Mb) was recorded on chromosome 7 while the largest (3.54 Mb) was on chromosome 1. Some regions showed overlapping peaks between the two reciprocal comparisons but the AQVs were not significant such as in chromosomes 9, 10 and 12. Candidate genes located under the significant top marker-bins were obtained from the published potato genome (Potato Genome Sequencing Consortium 2011; Sharma et al. 2013) and are listed in Table S6.

**Table 4:** Whole-exome capture sequencing read mapping and coverage (read depth) summary.

Sample	Total reads	Total mapped reads	Mean target coverage	Median target coverage
99FT1b5	186227992	142132331	101	93
HB171(13)	171371946	130475238	95	86
Dorm_t46_Bulk_1	342822138	228868401	137	127
Dorm_t46_Bulk_2	367288044	246182191	143	132
DM^a^	303225874	234258485	206	198

^a^Doubled monoploid *Solanum tuberosum* group Phureja DM1-3 516 R44 clone.

**Table 5:** Genomic coordinates of significant QTL regions detected by WEC-coupled BSA (BSA-QTL) for sprout growth at 46 days (t46) post harvest.

Chromosome	BSA-QTL start (Mb)	BSA-QTL end (Mb)	BSA-QTL size (Mb)
1	69.3	74.1	4.8
2	22.4	31.4	9
3	39.5	46.2	6.7
4	61.2	66.6	5.4
5	31.6	44	12.4
6	0.1	49.4	49.3
7	50.8	51	0.2
8	39.5	40	0.5

**Table 6:** Genomic coordinates of most significant marker-bin in BSA-QTL regions.

Chromosome	Marker-bin start (Mb)	Marker-bin end (Mb)	Marker-bin size (Mb)
1	69.93	73.47	3.54
2	24.40	25.45	1.05
3	42.24	42.52	0.29
4	61.44	61.69	0.25
5	43.39	43.89	0.49
6	0.09	0.26	0.17
6	3.21	3.51	0.29
7	50.85	51.00	0.15
8	39.58	39.98	0.40

## Discussion

In this paper, we report a detailed genetic study of potato tuber sprouting and dormancy release, assessed using tuber sprout growth. Our genetic material was a highly polymorphic diploid potato population derived from a cross between two very heterozygous parents. The population is somewhat unusual, showing a low average level of tuber dormancy and being uniformly very late maturing. Previous QTL analyses have revealed no genetic variation for foliage maturity at the well-described earliness locus on chromosome 5 that can often confound complex trait analysis (Kloosterman et al. 2013). Our study used a pre-existing SNP map (Prashar et al. 2014), which was extended to include a larger number of progeny clones (from 186 to 249). The number of mapped loci was increased to 3076 due to the inclusion of ‘nearly co-segregating’ markers omitted from the previously published version of the linkage map (Prashar et al. 2014). This increase in population size and marker number only led to a very slight (~3%) increase in overall map length, which suggests that both linkage maps are highly accurate.

QTL analysis employed two different analytical tools, a non-parametric method (KW) using MapQTL and a CIM approach using Genstat statistical software. The two methods were generally in very good agreement, both detecting QTL in the same approximate regions on chromosomes 2, 3, 4, 5 and 10. Taken together these results suggest that tuber dormancy break and sprout growth are genetically highly complex traits with many alleles segregating in a diploid population that differ in both the direction of the effect as well as the magnitude on the trait assessed.

As well as a conventional QTL approach, we also tested a pioneering BSA method, which makes use of a newly designed WEC platform for potato. This analysis involved WEC sequencing of DNA from phenotypic bulks displaying extreme sprout-growth phenotypes selected from the t46 sprout-growth data. The combined BSA and WEC approach identified QTL on chromosomes 1–8, the majority of which corroborated the QTL locations observed in the CIM-QTL analyses (Fig. 5). Moreover, the WEC-coupled BSA approach served to narrow the sprout-growth QTL to further smaller genomic regions ranging from 0.15 Mb for chromosome 7 QTL to 3.54 Mb for the QTL on chromosome 1 (Table 6), thereby, increasing precision and resolution of genetic studies previously not achievable in potato.

To illustrate the utility of integrating the conventional QTL and WEC-coupled BSA methods for genetic analyses, QTL regions identified using both approaches were subjected to further detailed evaluation. A candidate genomic-segment spanning ~3.2 Mb was identified in the CIM-QTL region on chromosome 3 near a SNP marker (c2_52371) based on ‘1-LOD drop’ threshold. This candidate region was further narrowed down to only 0.29 Mb based on the genomic coordinates inferred from the most significant marker-bin observed on chromosome 3 BSA-QTL. The protein-coding content in this short region containing 20 genes was inspected carefully and a *CENTRORADIALIS* gene (*StCEN*), which maps to 42.5 Mb on chromosome 3 (Table S6), was identified as a candidate gene for the causative factor underlying the QTL based on the known effects of *TFL1/CEN* homologues on bud maturation and outgrowth in several species (Mohamed et al. 2010; Varkonyi-Gasic et al. 2013). A transgenic approach was used to manipulate the expression level of the *TFL1*/*CEN* in a potato tetraploid genotype (cv. Désirée) (Morris et al. 2019). A clear effect of *TFL1*/*CEN* expression manipulation was demonstrated, with decreased expression levels associated with an increased rate of tuber sprout growth in storage, and over-expressing lines showing a lower rate of sprout growth than controls that occurred independently of changes in tuber endodormancy characteristics.

Only a few detailed genetical studies purporting to the genetics of potato tuber dormancy and sprout growth have been reported, although to the best of our knowledge, none of the previous studies have performed genetic analysis on tuber sprout-growth rates. One of the earliest such studies (Freyre et al. 1994) using a diploid population reports QTL effects on several chromosomes (2, 3, 4, 5, 7, 8) all of which are common to the findings from this study. However, the very poor resolution of the genetic map in the previous study makes detailed comparisons with this study impossible and also due to the lack of sequence-tagged markers correct orientation of the reported genetic maps cannot be ascertained. In a later study (van den Berg et al. 1996) that also suffered from poor map resolution, there were also dormancy QTL detected on chromosomes 1, 2, 3, 4, 5, 8, 9, 10 and 11, of which all but chromosome 9 QTL find similarities with those observed here. Despite the scant marker distribution in the older studies, the QTL reported in the present study appear to be in approximately the same locations as those detected in the earlier two publications, that is towards the ‘top’ of chromosomes 2 and 3 after accounting for possible reverse genetic orientation for these chromosomes reported by Freyre et al. (1994) and towards ‘bottom’ of chromosomes 4, 5, 8 and 10; locations for chromosome 1 (van den Berg et al. 1996) and 7 (Freyre et al. 1994) QTL were unresolved or not clear in the reported studies, so it is tantalising to speculate that these effects may share common origins. A much more recent potato dormancy study (Bisognin et al. 2018) also reports a complex pattern of genetic effects mapping to seven potato chromosomes (2, 3, 5, 6, 7, 9 and 11). There is a high likelihood that the common QTL effects detected on chromosomes 2, 3, 5, 6 and 7 in the previous publication may be syntenic with effects reported here at similar map positions. The current and previously reported studies involve crosses derived from very diverse parental material, some even from wild species, and as such might be expected to show different QTL. However, several QTL are close enough to suggest that they are likely of the same origin. In particular, the mapping population used by Freyre et al. (1994) is derived from a ‘tuberosum hybrid’ (female) and ‘*S. phureja*’ (male) cross and that deployed by Bisognin et al. (2018) contained *S. tuberosum* and *S. phureja* in the pedigree suggesting that QTL observed in these studies have higher correspondence and transferability with those observed in the current study.

It is worth noting that only one of the previous studies (van den Berg et al. 1996) was performed on data from 2 years and that study used unequal number of clones in both years while the other studies were performed on single year data only. Also, Bisognin et al. (2018) reported QTL for ‘days-to-apical-dominance-release’ and ‘days-to-dormancy-release’ phenotypes while the other two only for the latter trait. The current study deploys a robust 2-year data set for six time-point traits and the similarity of most of the QTL validates the use of tuber sprout growth as a proxy for tuber dormancy. Our findings suggest that tuber dormancy and sprout growth are highly complex developmental processes, under the control of several genes. Encouragingly our study compares well with the very few other published studies in potato, suggesting that the loci discovered that are in common between the various studies may be the main ones controlling these highly important traits. The high level of complexity of the traits studied here suggest that use of a genomic selection approach may be needed for making progress in breeding for longer tuber dormancy and slower sprout growth. However, such a strategy would need to take account of the many other important traits that could not be ignored in any potato breeding programme. Alternatively, it may be possible to modify tuber dormancy and sprouting traits using biotechnological approaches, but this will require a greater knowledge of the causative genes involved in the trait to become a realistic option.

The WEC-BSA analysis has identified a large number (over 500) of candidate genes underlying eight QTL regions. This raises questions about the identification of the causative genes underlying complex trait QTLs such as those reported here. Fine mapping approaches have been problematic in diploid potato due to the need to use two highly heterozygous parents for the generation of mapping crosses. Such crosses lead to complex segregation patterns and fairly low accuracies of QTL localisation. Supplementary approaches are required to refine the list of candidate genes within such QTL regions as are reported here. Network modelling represents a powerful tool that can unravel properties of complex biological systems, and this approach could be applied to potato dormancy and sprouting from time-resolved transcriptomics data. Indeed, such an approach has recently been used to understand immune signalling in potato (Ramsak et al. 2018). Previously, we have used a bulked transcriptomic approach to identify novel candidate genes in potato (Campbell et al. 2014) and a similar approach could also be used to dissect the sprouting QTL reported here.

Sequence-based genotyping is now the preferred method for high-throughput low-cost genotyping. The BSA approach implemented here benefited from several advantages offered by use of ‘open’ genotyping platforms, which provide greater flexibility, scalability and the capability to scan polymorphisms without a priori knowledge (Sharma and Bryan 2017). We have utilised a BSA strategy using a novel WEC platform to saturate the genome with SNP variants present in coding regions of the potato genome. The effective SNP density reached for the BSA analysis was 17,156 SNPs per Mb of the WEC target region. In contrast, the Illumina Infinium 8k SNPs provided a density of only 365 SNPs per Mb in the genic regions covered by this platform (Felcher et al. 2012). The Illumina 8k SNP array is based on potato transcriptome sequencing, but covers only 3591 of the 39,031 protein-coding genes (Felcher et al. 2012) predicted by Potato Genome Sequencing Consortium (2011). The much higher SNP density achieved through WEC genotyping compared with the SNP array potentially contributed to increasing the saturation of QTL regions with markers, further enhancing the scope for identifying causal gene(s). Another significant advantage offered by the BSA approach is the need to genotype individuals from only the extreme tails of the trait distribution especially where the number of progeny clones is very large. Genetic analysis of larger populations enhances mapping resolution through increased number of recombination events and enhances the power to detect QTL and perform fine mapping of loci underlying them. The use of significantly sized bulks (in this case 20 plants) is a pragmatic method for ‘capturing’ recombination events in QTL regions, thus making the BSA approach beneficial in terms of cost and effort. In addition to potato, the findings we report here have significant implications for the breeding and post-harvest handling of other root, bulb and tuber crops (such as sugar beet, onion and yam) that are required to undergo long-term storage.

## Supplementary information

suppInfo_FigureS1_plus_Tables_S1toS5

TableS6_BSA_QTL_geneList

## Data Availability

The genotype and phenotype information used for this study is available on the Dryad data repository (10.5061/dryad.2rbnzs7nt). Sequence data have been deposited in the European Nucleotide Archive (ENA) at EMBL-EBI under accession number PRJEB46000.
