# Supplementary material for: Combining conventional QTL analysis and whole-exome capture-based bulk-segregant analysis provides new genetic insights into tuber sprout elongation and dormancy release in a diploid potato population
Source: Heredity (Edinb). 2021 Jul 30;127(3):253–65. doi: 10.1038/s41437-021-00459-0 (PMC8405706; doi:10.1038/s41437-021-00459-0)
Supplement: Supplementary file 1 — suppInfo_FigureS1_plus_Tables_S1toS5 [file 41437_2021_459_MOESM1_ESM.pdf]

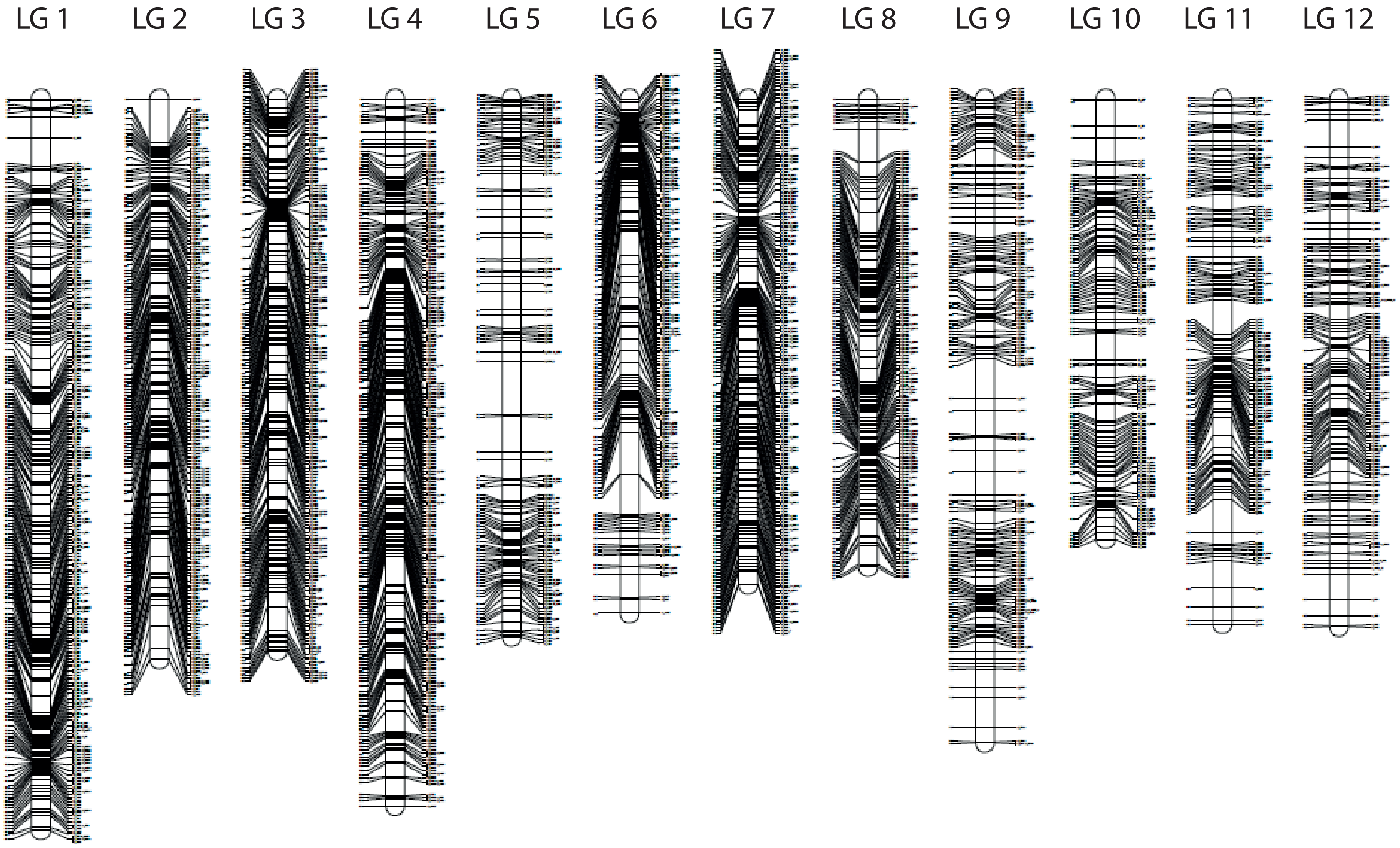

Figure S1: 06H1 linkage map constructed using 249 progeny clones and 3052 marker loci filtered from Illumina Infinium 8k Potato SNP Array (HAMILTON et al. 2011; FELCHER et al. 2012).

Table S1: List of 06H1 clones included in 'low' and 'high' tuber sprout growth rate bulks for performing bulk-segregant analysis.

06H1 Progeny Clones	Mean Tuber Sprout Length (mm; time-point t46)	bulkType
06.H.1 A 427	1.1	Low Sprout Growth Rate
06.H.1 A 110	1.0	Low Sprout Growth Rate
06.H.1 A 393	0.8	Low Sprout Growth Rate
06.H.1 A 66	0.9	Low Sprout Growth Rate
06.H.1 A 233	1.0	Low Sprout Growth Rate
06.H.1 A 2	1.1	Low Sprout Growth Rate
06.H.1 A 243	1.4	Low Sprout Growth Rate
06.H.1 A 389	1.8	Low Sprout Growth Rate
06.H.1 A 314	1.4	Low Sprout Growth Rate
06.H.1 A 101	1.3	Low Sprout Growth Rate
06.H.1 A 400	1.8	Low Sprout Growth Rate
06.H.1 A 27	1.2	Low Sprout Growth Rate
06.H.1 A 107	2.7	Low Sprout Growth Rate
06.H.1 A 414	1.6	Low Sprout Growth Rate
06.H.1 A 9	2.9	Low Sprout Growth Rate
06.H.1 A 117	1.5	Low Sprout Growth Rate
06.H.1 A 383	1.6	Low Sprout Growth Rate
06.H.1 A 223	2.4	Low Sprout Growth Rate
06.H.1 A 279	1.9	Low Sprout Growth Rate
06.H.1 A 315	2.6	Low Sprout Growth Rate
06.H.1 A 152	16.7	High Sprout Growth Rate
06.H.1 A 92	20.2	High Sprout Growth Rate
06.H.1 A 10	19.0	High Sprout Growth Rate
06.H.1 A 200	16.8	High Sprout Growth Rate
06.H.1 A 173	19.4	High Sprout Growth Rate
06.H.1 A 369	16.6	High Sprout Growth Rate
06.H.1 A 258	18.6	High Sprout Growth Rate
06.H.1 A 407	16.9	High Sprout Growth Rate
06.H.1 A 422	15.9	High Sprout Growth Rate
06.H.1 A 302	18.3	High Sprout Growth Rate
06.H.1 A 69	24.9	High Sprout Growth Rate
06.H.1 A 355	16.2	High Sprout Growth Rate
06.H.1 A 155	19.3	High Sprout Growth Rate
06.H.1 A 353	22.0	High Sprout Growth Rate
06.H.1 A 80	19.0	High Sprout Growth Rate
06.H.1 A 291	20.5	High Sprout Growth Rate
06.H.1 A 241	27.5	High Sprout Growth Rate
06.H.1 A 287	24.7	High Sprout Growth Rate
06.H.1 A 65	22.9	High Sprout Growth Rate
06.H.1 A 384	31.1	High Sprout Growth Rate

Table S2: 06H1 genetic linkage map statistics.

Chromosome	Map Length (cM)	Number of Markers
1	87.01	370
2	61.8	304
3	65.67	316
4	70.98	351
5	64.01	161
6	61.16	258
7	57.77	310
8	55.65	230
9	76.58	208
10	52.42	184
11	62.52	207
12	62.84	177
**TOTAL**	**778.41**	**3076**

Table S3: Correlations between trait means for all time-point measurements as well as BETA and KAPPA.

		2014							
		t4	t18	t32	t46	t60	t74	beta	kappa
2013	t4		0.76	0.63	0.54	0.48	0.42	-0.52	-0.07
	t18	0.88		0.90	0.78	0.70	0.57	-0.77	-0.13
	t32	0.78	0.94		0.90	0.85	0.73	-0.85	-0.04
	t46	0.72	0.89	0.95		0.93	0.86	-0.82	0.15
	t60	0.66	0.83	0.91	0.95		0.92	-0.76	0.29
	t74	0.59	0.72	0.77	0.84	0.90		-0.56	0.55
	beta	-0.67	-0.78	-0.81	-0.77	-0.69	-0.47		0.35
	kappa	0.13	-0.08	-0.03	0.08	0.22	0.49	0.52	

Table S4: Results of non-parametric QTL analysis from MapQTL6 (Kruskal-Wallis analysis). Values provided in the body of the table are K values that exceed the significance level based on a permutation test (K > 13).

	Trait locus	Chromosome 2 12.84 cM		Chromosome 3 3.27 cM		Chromosome 3 19.83 cM		Chromosome 4 38.97 cM		Chromosome 5 60.76 cM		Chromosome 10 41.99 cM	
	**Marker (type)**	**c1_9719 (hkxhk)**		**c2_17218 (nnxnp)**		**c2_52371 (nnxnp)**		**c1_11758 (hkxhk)**		**c2_8515 (nnxnp)**		**c2_54862 (nnxnp)**	
	**Year**	**2013**	**2014**	**2013**	**2014**	**2013**	**2014**	**2013**	**2014**	**2013**	**2014**	**2013**	**2014**
**Time-point traits**	**t4**	-	-	30.98	14.7	34.1	19.36	13.37	13.34	-	-	-	-
	**t18**	22.35	-	35.44	28.52	37.83	41.7	14.16	20.04	18.33	14.13	-	-
	**t32**	32.82	17.99	37.07	30.67	36.19	41.56	16.35	13.43	21.45	19.88	-	-
	**t46**	35.12	19.52	32.89	35.87	32.16	39.69	17.04	17.08	21.28	25.16	19.27	13.27
	**t60**	36.53	27.08	31.53	29.53	29.2	31.26	13.17	19.4	20.02	28.26	27.08	20
	**t74**	38.2	31.41	26.44	25.06	24.29	31.58	20.44	15.39	21.09	23.85	25.68	18.83
**Modelled Estimates**													
	**BETA**	18.25	-	37.41	20.09	34.03	35.09	-	-	14.02	-	-	-
	**KAPPA**	-	24.37	-	-	-	-	-	-	-	-	-	20.54

Table S5: Average-Q-value cut-off limits (at 5% FDR threshold) calculated separately for each individual chromosome.

Chromosome	Mean_Q	Stddev_Q	Average_Q_value*	BSA Comparison
1	6.0	8.8	24.9	LowSproutGrowth VS HighSproutGrowth
2	6.2	11.9	31.8	LowSproutGrowth VS HighSproutGrowth
3	14.6	26.7	79.3	LowSproutGrowth VS HighSproutGrowth
4	9.9	19.8	59.2	LowSproutGrowth VS HighSproutGrowth
5	4.1	6.9	19.4	LowSproutGrowth VS HighSproutGrowth
6	3.2	6.1	14.9	LowSproutGrowth VS HighSproutGrowth
7	3.5	8.0	21.9	LowSproutGrowth VS HighSproutGrowth
8	7.3	10.4	31.1	LowSproutGrowth VS HighSproutGrowth
9	7.7	11.0	35.2	LowSproutGrowth VS HighSproutGrowth
10	21.1	30.5	105.7	LowSproutGrowth VS HighSproutGrowth
11	2.3	4.7	12.3	LowSproutGrowth VS HighSproutGrowth
12	7.6	10.9	35.0	LowSproutGrowth VS HighSproutGrowth
1	9.0	13.4	41.1	HighSproutGrowth VS LowSproutGrowth
2	6.1	12.2	32.2	HighSproutGrowth VS LowSproutGrowth
3	7.0	9.8	27.9	HighSproutGrowth VS LowSproutGrowth
4	9.4	15.9	44.8	HighSproutGrowth VS LowSproutGrowth
5	3.1	4.8	13.0	HighSproutGrowth VS LowSproutGrowth
6	3.1	5.3	13.1	HighSproutGrowth VS LowSproutGrowth
7	3.1	6.4	16.9	HighSproutGrowth VS LowSproutGrowth
8	6.4	8.7	26.9	HighSproutGrowth VS LowSproutGrowth
9	14.5	26.9	91.6	HighSproutGrowth VS LowSproutGrowth
10	20.0	26.1	86.4	HighSproutGrowth VS LowSproutGrowth
11	2.7	9.7	26.7	HighSproutGrowth VS LowSproutGrowth
12	12.1	18.5	60.7	HighSproutGrowth VS LowSproutGrowth

** Average Q value threshold at 5% FDR significance level*
